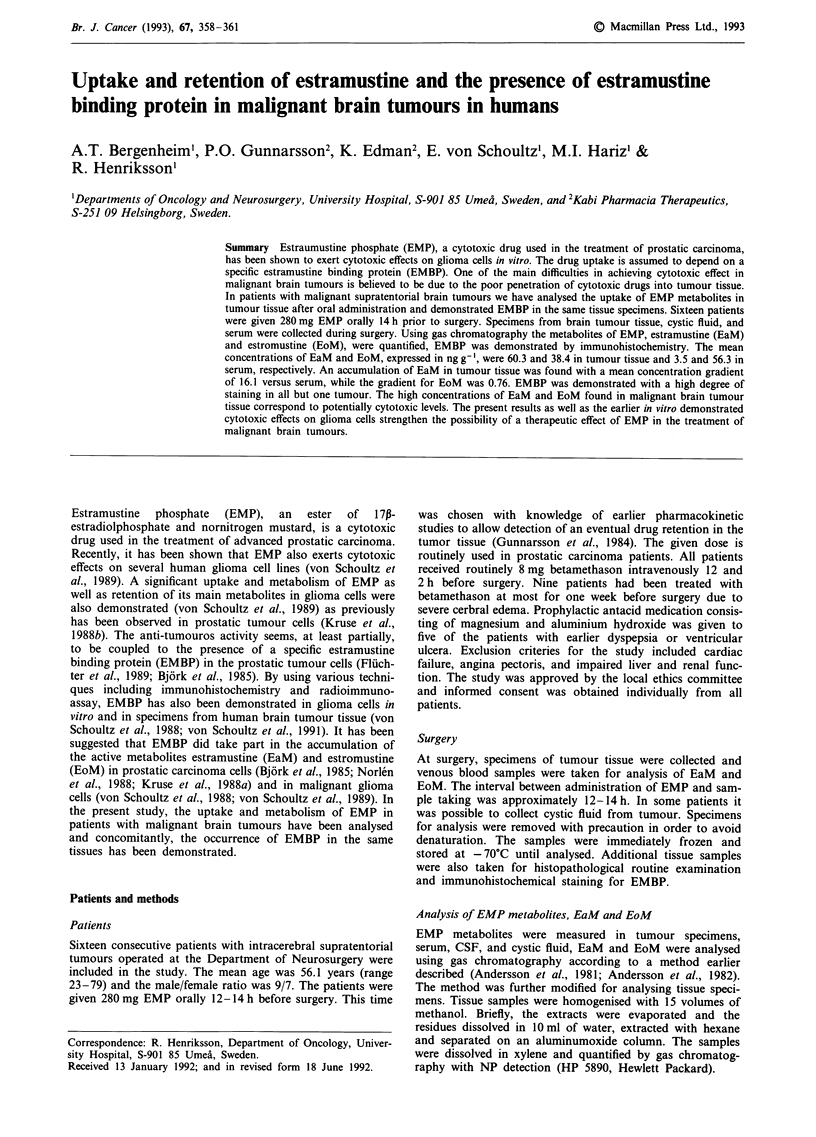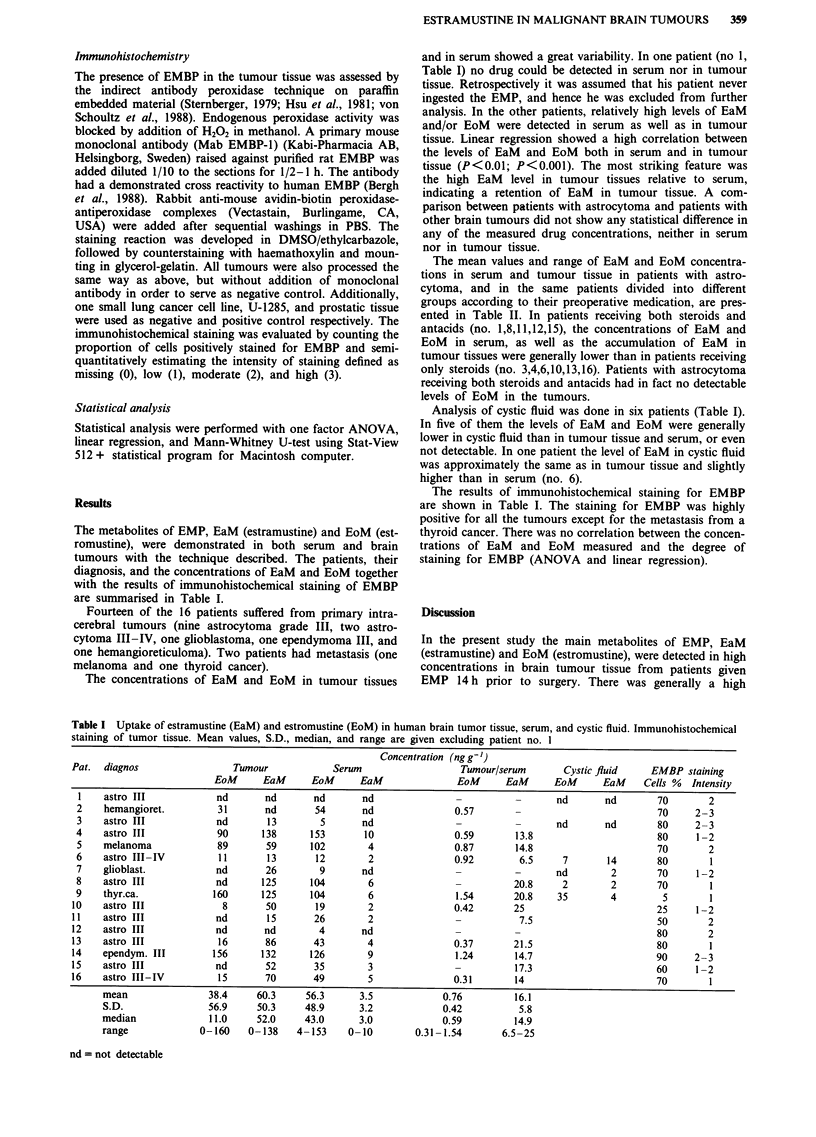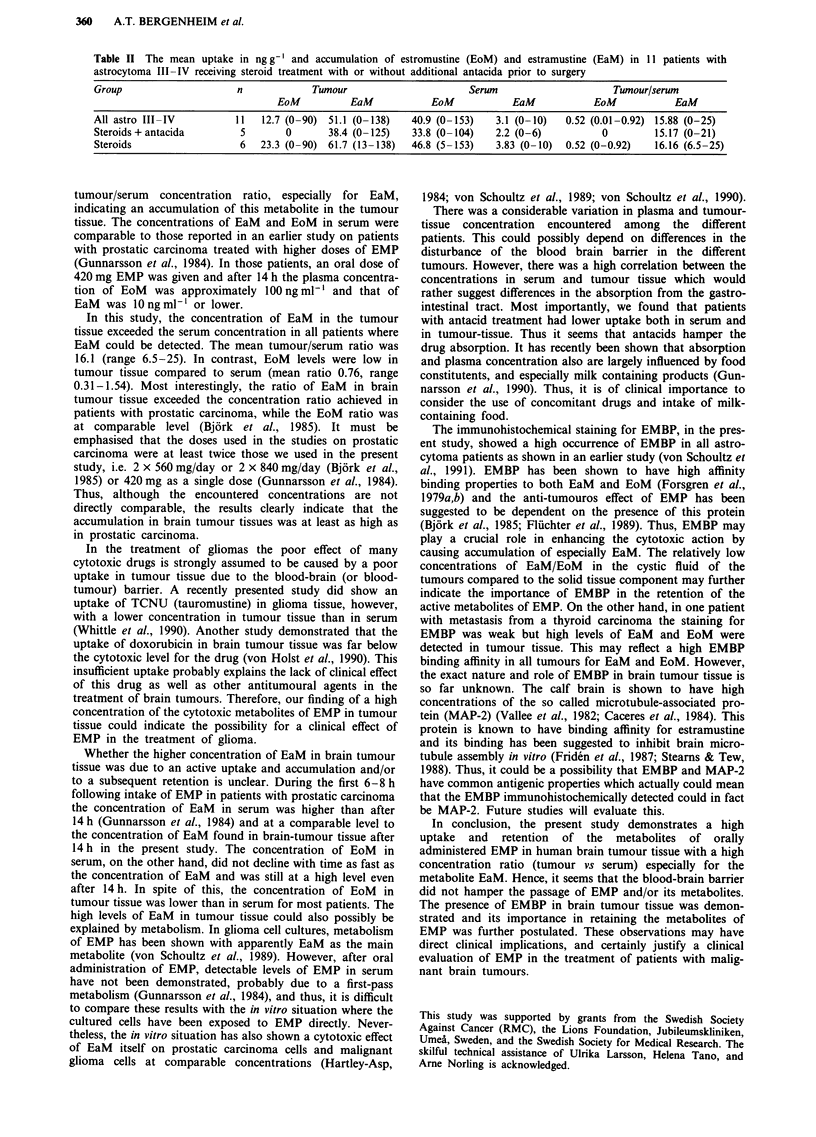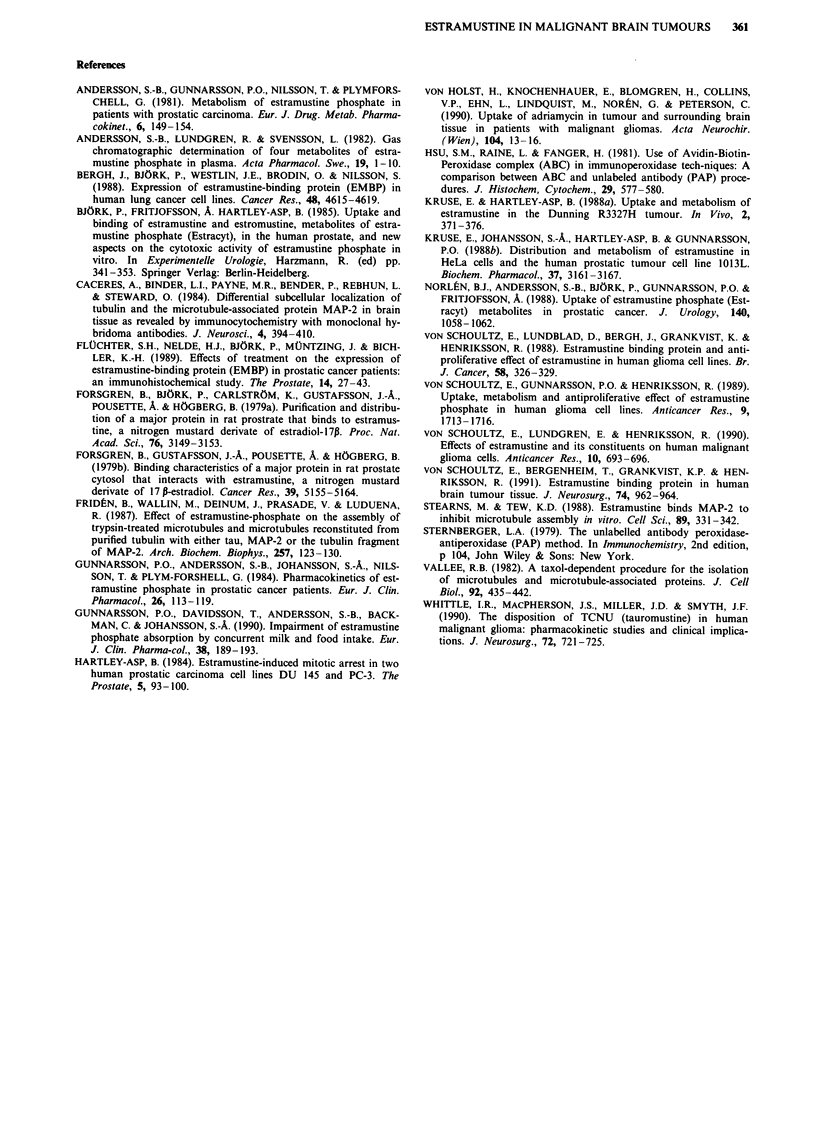# Uptake and retention of estramustine and the presence of estramustine binding protein in malignant brain tumours in humans.

**DOI:** 10.1038/bjc.1993.65

**Published:** 1993-02

**Authors:** A. T. Bergenheim, P. O. Gunnarsson, K. Edman, E. von Schoultz, M. I. Hariz, R. Henriksson

**Affiliations:** Department of Oncology, University Hospital, Umeå, Sweden.

## Abstract

Estraumustine phosphate (EMP), a cytotoxic drug used in the treatment of prostatic carcinoma, has been shown to exert cytotoxic effects on glioma cells in vitro. The drug uptake is assumed to depend on a specific estramustine binding protein (EMBP). One of the main difficulties in achieving cytotoxic effect in malignant brain tumours is believed to be due to the poor penetration of cytotoxic drugs into tumour tissue. In patients with malignant supratentorial brain tumours we have analysed the uptake of EMP metabolites in tumour tissue after oral administration and demonstrated EMBP in the same tissue specimens. Sixteen patients were given 280 mg EMP orally 14 h prior to surgery. Specimens from brain tumour tissue, cystic fluid, and serum were collected during surgery. Using gas chromatography the metabolites of EMP, estramustine (EaM) and estromustine (EoM), were quantified, EMBP was demonstrated by immunohistochemistry. The mean concentrations of EaM and EoM, expressed in ng g-1, were 60.3 and 38.4 in tumour tissue and 3.5 and 56.3 in serum, respectively. An accumulation of EaM in tumour tissue was found with a mean concentration gradient of 16.1 versus serum, while the gradient for EoM was 0.76. EMBP was demonstrated with a high degree of staining in all but one tumour. The high concentrations of EaM and EoM found in malignant brain tumour tissue correspond to potentially cytotoxic levels. The present results as well as the earlier in vitro demonstrated cytotoxic effects on glioma cells strengthen the possibility of a therapeutic effect of EMP in the treatment of malignant brain tumours.


Br. J. Cancer (1993), 67, 358–361 © Macmillan Press Ltd., 1993

### Uptake and retention of estramustine and the presence of estramustine binding protein in malignant brain tumours in humans

A.T. Bergenheim[1], P.O. Gunnarsson[2], K. Edman[2], E. von Schoultz[1], M.I. Hariz[1] & R. Henriksson[1]

[1]*Departments of Oncology and Neurosurgery, University Hospital, S-901 85 Umeå, Sweden, and* [2]*Kabi Pharmacia Therapeutics, S-251 09 Helsingborg, Sweden.*

**Summary** Estraumustine phosphate (EMP), a cytotoxic drug used in the treatment of prostatic carcinoma, has been shown to exert cytotoxic effects on glioma cells *in vitro*. The drug uptake is assumed to depend on a specific estramustine binding protein (EMBP). One of the main difficulties in achieving cytotoxic effect in malignant brain tumours is believed to be due to the poor penetration of cytotoxic drugs into tumour tissue. In patients with malignant supratentorial brain tumours we have analysed the uptake of EMP metabolites in tumour tissue after oral administration and demonstrated EMBP in the same tissue specimens. Sixteen patients were given 280 mg EMP orally 14 h prior to surgery. Specimens from brain tumour tissue, cystic fluid, and serum were collected during surgery. Using gas chromatography the metabolites of EMP, estramustine (EaM) and estromustine (EoM), were quantified, EMBP was demonstrated by immunohistochemistry. The mean concentrations of EaM and EoM, expressed in ng $g^{-1}$, were 60.3 and 38.4 in tumour tissue and 3.5 and 56.3 in serum, respectively. An accumulation of EaM in tumour tissue was found with a mean concentration gradient of 16.1 versus serum, while the gradient for EoM was 0.76. EMBP was demonstrated with a high degree of staining in all but one tumour. The high concentrations of EaM and EoM found in malignant brain tumour tissue correspond to potentially cytotoxic levels. The present results as well as the earlier *in vitro* demonstrated cytotoxic effects on glioma cells strengthen the possibility of a therapeutic effect of EMP in the treatment of malignant brain tumours.

Estramustine phosphate (EMP), an ester of 17β-estradiolphosphate and nornitrogen mustard, is a cytotoxic drug used in the treatment of advanced prostatic carcinoma. Recently, it has been shown that EMP also exerts cytotoxic effects on several human glioma cell lines (von Schoultz *et al*., 1989). A significant uptake and metabolism of EMP as well as retention of its main metabolites in glioma cells were also demonstrated (von Schoultz *et al*., 1989) as previously has been observed in prostatic tumour cells (Kruse *et al*., 1988*b*). The anti-tumouros activity seems, at least partially, to be coupled to the presence of a specific estramustine binding protein (EMBP) in the prostatic tumour cells (Flüchter *et al*., 1989; Björk *et al*., 1985). By using various techniques including immunohistochemistry and radioimmunoassay, EMBP has also been demonstrated in glioma cells *in vitro* and in specimens from human brain tumour tissue (von Schoultz *et al*., 1988; von Schoultz *et al*., 1991). It has been suggested that EMBP did take part in the accumulation of the active metabolites estramustine (EaM) and estromustine (EoM) in prostatic carcinoma cells (Björk *et al*., 1985; Norlén *et al*., 1988; Kruse *et al*., 1988*a*) and in malignant glioma cells (von Schoultz *et al*., 1988; von Schoultz *et al*., 1989). In the present study, the uptake and metabolism of EMP in patients with malignant brain tumours have been analysed and concomitantly, the occurrence of EMBP in the same tissues has been demonstrated.

### Patients and methods

### *Patients*

Sixteen consecutive patients with intracerebral supratentorial tumours operated at the Department of Neurosurgery were included in the study. The mean age was 56.1 years (range 23–79) and the male/female ratio was 9/7. The patients were given 280 mg EMP orally 12–14 h before surgery. This time was chosen with knowledge of earlier pharmacokinetic studies to allow detection of an eventual drug retention in the tumor tissue (Gunnarsson *et al*., 1984). The given dose is routinely used in prostatic carcinoma patients. All patients received routinely 8 mg betamethason intravenously 12 and 2 h before surgery. Nine patients had been treated with betamethason at most for one week before surgery due to severe cerebral edema. Prophylactic antacid medication consisting of magnesium and aluminium hydroxide was given to five of the patients with earlier dyspepsia or ventricular ulcera. Exclusion criteria for the study included cardiac failure, angina pectoris, and impaired liver and renal function. The study was approved by the local ethics committee and informed consent was obtained individually from all patients.

### *Surgery*

At surgery, specimens of tumour tissue were collected and venous blood samples were taken for analysis of EaM and EoM. The interval between administration of EMP and sample taking was approximately 12–14 h. In some patients it was possible to collect cystic fluid from tumour. Specimens for analysis were removed with precaution in order to avoid denaturation. The samples were immediately frozen and stored at −70°C until analysed. Additional tissue samples were also taken for histopathological routine examination and immunohistochemical staining for EMBP.

### *Analysis of EMP metabolites, EaM and EoM*

EMP metabolites were measured in tumour specimens, serum, CSF, and cystic fluid, EaM and EoM were analysed using gas chromatography according to a method earlier described (Andersson *et al*., 1981; Andersson *et al*., 1982). The method was further modified for analysing tissue specimens. Tissue samples were homogenised with 15 volumes of methanol. Briefly, the extracts were evaporated and the residues dissolved in 10 ml of water, extracted with hexane and separated on an aluminumoxide column. The samples were dissolved in xylene and quantified by gas chromatography with NP detection (HP 5890, Hewlett Packard).

Correspondence: R. Henriksson, Department of Oncology, University Hospital, S-901 85 Umeå, Sweden.
Received 13 January 1992; and in revised form 18 June 1992.

### *Immunohistochemistry*

The presence of EMBP in the tumour tissue was assessed by the indirect antibody peroxidase technique on paraffin embedded material (Sternberger, 1979; Hsu *et al.*, 1981; von Schoultz *et al.*, 1988). Endogenous peroxidase activity was blocked by addition of $H_2O_2$ in methanol. A primary mouse monoclonal antibody (Mab EMBP-1) (Kabi-Pharmacia AB, Helsingborg, Sweden) raised against purified rat EMBP was added diluted 1/10 to the sections for 1/2–1 h. The antibody had a demonstrated cross reactivity to human EMBP (Bergh *et al.*, 1988). Rabbit anti-mouse avidin-biotin peroxidase-antiperoxidase complexes (Vectastain, Burlingame, CA, USA) were added after sequential washings in PBS. The staining reaction was developed in DMSO/ethylcarbazole, followed by counterstaining with haemathoxylin and mounting in glycerol-gelatin. All tumours were also processed the same way as above, but without addition of monoclonal antibody in order to serve as negative control. Additionally, one small lung cancer cell line, U-1285, and prostatic tissue were used as negative and positive control respectively. The immunohistochemical staining was evaluated by counting the proportion of cells positively stained for EMBP and semi-quantitatively estimating the intensity of staining defined as missing (0), low (1), moderate (2), and high (3).

### *Statistical analysis*

Statistical analysis were performed with one factor ANOVA, linear regression, and Mann-Whitney U-test using Stat-View 512 + statistical program for Macintosh computer.

### Results

The metabolites of EMP, EaM (estramustine) and EoM (estromustine), were demonstrated in both serum and brain tumours with the technique described. The patients, their diagnosis, and the concentrations of EaM and EoM together with the results of immunohistochemical staining of EMBP are summarised in Table I.

Fourteen of the 16 patients suffered from primary intracerebral tumours (nine astrocytoma grade III, two astrocytoma III–IV, one glioblastoma, one ependymoma III, and one hemangioreticuloma). Two patients had metastasis (one melanoma and one thyroid cancer).

The concentrations of EaM and EoM in tumour tissues and in serum showed a great variability. In one patient (no 1, Table I) no drug could be detected in serum nor in tumour tissue. Retrospectively it was assumed that his patient never ingested the EMP, and hence he was excluded from further analysis. In the other patients, relatively high levels of EaM and/or EoM were detected in serum as well as in tumour tissue. Linear regression showed a high correlation between the levels of EaM and EoM both in serum and in tumour tissue ($P < 0.01$; $P < 0.001$). The most striking feature was the high EaM level in tumour tissues relative to serum, indicating a retention of EaM in tumour tissue. A comparison between patients with astrocytoma and patients with other brain tumours did not show any statistical difference in any of the measured drug concentrations, neither in serum nor in tumour tissue.

The mean values and range of EaM and EoM concentrations in serum and tumour tissue in patients with astrocytoma, and in the same patients divided into different groups according to their preoperative medication, are presented in Table II. In patients receiving both steroids and antacids (no. 1,8,11,12,15), the concentrations of EaM and EoM in serum, as well as the accumulation of EaM in tumour tissues were generally lower than in patients receiving only steroids (no. 3,4,6,10,13,16). Patients with astrocytoma receiving both steroids and antacids had in fact no detectable levels of EoM in the tumours.

Analysis of cystic fluid was done in six patients (Table I). In five of them the levels of EaM and EoM were generally lower in cystic fluid than in tumour tissue and serum, or even not detectable. In one patient the level of EaM in cystic fluid was approximately the same as in tumour tissue and slightly higher than in serum (no. 6).

The results of immunohistochemical staining for EMBP are shown in Table I. The staining for EMBP was highly positive for all the tumours except for the metastasis from a thyroid cancer. There was no correlation between the concentrations of EaM and EoM measured and the degree of staining for EMBP (ANOVA and linear regression).

### Discussion

In the present study the main metabolites of EMP, EaM (estramustine) and EoM (estromustine), were detected in high concentrations in brain tumour tissue from patients given EMP 14 h prior to surgery. There was generally a high

**Table I** Uptake of estramustine (EaM) and estromustine (EoM) in human brain tumor tissue, serum, and cystic fluid. Immunohistochemical staining of tumor tissue. Mean values, S.D., median, and range are given excluding patient no. 1

| | | Concentration ($ng\ g^{-1}$) | | | | | | | | EMBP staining | |
|---|---|---|---|---|---|---|---|---|---|---|---|
| *Pat.* | *diagnos* | *Tumour* | | *Serum* | | *Tumour/serum* | | *Cystic fluid* | | | |
| | | *EoM* | *EaM* | *EoM* | *EaM* | *EoM* | *EaM* | *EoM* | *EaM* | *Cells %* | *Intensity* |
| 1 | astro III | nd | nd | nd | nd | – | – | nd | nd | 70 | 2 |
| 2 | hemangioret. | 31 | nd | 54 | nd | 0.57 | – | | | 70 | 2–3 |
| 3 | astro III | nd | 13 | 5 | nd | – | – | nd | nd | 80 | 2–3 |
| 4 | astro III | 90 | 138 | 153 | 10 | 0.59 | 13.8 | | | 80 | 1–2 |
| 5 | melanoma | 89 | 59 | 102 | 4 | 0.87 | 14.8 | | | 70 | 2 |
| 6 | astro III–IV | 11 | 13 | 12 | 2 | 0.92 | 6.5 | 7 | 14 | 80 | 1 |
| 7 | glioblast. | nd | 26 | 9 | nd | – | – | nd | 2 | 70 | 1–2 |
| 8 | astro III | nd | 125 | 104 | 6 | – | 20.8 | 2 | 2 | 70 | 1 |
| 9 | thyr.ca. | 160 | 125 | 104 | 6 | 1.54 | 20.8 | 35 | 4 | 5 | 1 |
| 10 | astro III | 8 | 50 | 19 | 2 | 0.42 | 25 | | | 25 | 1–2 |
| 11 | astro III | nd | 15 | 26 | 2 | – | 7.5 | | | 50 | 2 |
| 12 | astro III | nd | nd | 4 | nd | – | – | | | 80 | 2 |
| 13 | astro III | 16 | 86 | 43 | 4 | 0.37 | 21.5 | | | 80 | 1 |
| 14 | ependym. III | 156 | 132 | 126 | 9 | 1.24 | 14.7 | | | 90 | 2–3 |
| 15 | astro III | nd | 52 | 35 | 3 | – | 17.3 | | | 60 | 1–2 |
| 16 | astro III–IV | 15 | 70 | 49 | 5 | 0.31 | 14 | | | 70 | 1 |
| | mean | 38.4 | 60.3 | 56.3 | 3.5 | 0.76 | 16.1 | | | | |
| | S.D. | 56.9 | 50.3 | 48.9 | 3.2 | 0.42 | 5.8 | | | | |
| | median | 11.0 | 52.0 | 43.0 | 3.0 | 0.59 | 14.9 | | | | |
| | range | 0–160 | 0–138 | 4–153 | 0–10 | 0.31–1.54 | 6.5–25 | | | | |

nd = not detectable

**Table II** The mean uptake in ng g$^{-1}$ and accumulation of estromustine (EoM) and estramustine (EaM) in 11 patients with astrocytoma III–IV receiving steroid treatment with or without additional antacida prior to surgery

| *Group* | *n* | *Tumour* | | *Serum* | | *Tumour/serum* | |
|---|---|---|---|---|---|---|---|
| | | *EoM* | *EaM* | *EoM* | *EaM* | *EoM* | *EaM* |
| All astro III–IV | 11 | 12.7 (0–90) | 51.1 (0–138) | 40.9 (0–153) | 3.1 (0–10) | 0.52 (0.01–0.92) | 15.88 (0–25) |
| Steroids + antacida | 5 | 0 | 38.4 (0–125) | 33.8 (0–104) | 2.2 (0–6) | 0 | 15.17 (0–21) |
| Steroids | 6 | 23.3 (0–90) | 61.7 (13–138) | 46.8 (5–153) | 3.83 (0–10) | 0.52 (0–0.92) | 16.16 (6.5–25) |

tumour/serum concentration ratio, especially for EaM, indicating an accumulation of this metabolite in the tumour tissue. The concentrations of EaM and EoM in serum were comparable to those reported in an earlier study on patients with prostatic carcinoma treated with higher doses of EMP (Gunnarsson *et al.*, 1984). In those patients, an oral dose of 420 mg EMP was given and after 14 h the plasma concentration of EoM was approximately 100 ng ml$^{-1}$ and that of EaM was 10 ng ml$^{-1}$ or lower.

In this study, the concentration of EaM in the tumour tissue exceeded the serum concentration in all patients where EaM could be detected. The mean tumour/serum ratio was 16.1 (range 6.5–25). In contrast, EoM levels were low in tumour tissue compared to serum (mean ratio 0.76, range 0.31–1.54). Most interestingly, the ratio of EaM in brain tumour tissue exceeded the concentration ratio achieved in patients with prostatic carcinoma, while the EoM ratio was at comparable level (Björk *et al.*, 1985). It must be emphasised that the doses used in the studies on prostatic carcinoma were at least twice those we used in the present study, i.e. 2 × 560 mg/day or 2 × 840 mg/day (Björk *et al.*, 1985) or 420 mg as a single dose (Gunnarsson *et al.*, 1984). Thus, although the encountered concentrations are not directly comparable, the results clearly indicate that the accumulation in brain tumour tissues was at least as high as in prostatic carcinoma.

In the treatment of gliomas the poor effect of many cytotoxic drugs is strongly assumed to be caused by a poor uptake in tumour tissue due to the blood-brain (or blood-tumour) barrier. A recently presented study did show an uptake of TCNU (tauromustine) in glioma tissue, however, with a lower concentration in tumour tissue than in serum (Whittle *et al.*, 1990). Another study demonstrated that the uptake of doxorubicin in brain tumour tissue was far below the cytotoxic level for the drug (von Holst *et al.*, 1990). This insufficient uptake probably explains the lack of clinical effect of this drug as well as other antitumoural agents in the treatment of brain tumours. Therefore, our finding of a high concentration of the cytotoxic metabolites of EMP in tumour tissue could indicate the possibility for a clinical effect of EMP in the treatment of glioma.

Whether the higher concentration of EaM in brain tumour tissue was due to an active uptake and accumulation and/or to a subsequent retention is unclear. During the first 6–8 h following intake of EMP in patients with prostatic carcinoma the concentration of EaM in serum was higher than after 14 h (Gunnarsson *et al.*, 1984) and at a comparable level to the concentration of EaM found in brain-tumour tissue after 14 h in the present study. The concentration of EoM in serum, on the other hand, did not decline with time as fast as the concentration of EaM and was still at a high level even after 14 h. In spite of this, the concentration of EoM in tumour tissue was lower than in serum for most patients. The high levels of EaM in tumour tissue could also possibly be explained by metabolism. In glioma cell cultures, metabolism of EMP has been shown with apparently EaM as the main metabolite (von Schoultz *et al.*, 1989). However, after oral administration of EMP, detectable levels of EMP in serum have not been demonstrated, probably due to a first-pass metabolism (Gunnarsson *et al.*, 1984), and thus, it is difficult to compare these results with the *in vitro* situation where the cultured cells have been exposed to EMP directly. Nevertheless, the *in vitro* situation has also shown a cytotoxic effect of EaM itself on prostatic carcinoma cells and malignant glioma cells at comparable concentrations (Hartley-Asp, 1984; von Schoultz *et al.*, 1989; von Schoultz *et al.*, 1990).

There was a considerable variation in plasma and tumour-tissue concentration encountered among the different patients. This could possibly depend on differences in the disturbance of the blood brain barrier in the different tumours. However, there was a high correlation between the concentrations in serum and tumour tissue which would rather suggest differences in the absorption from the gastro-intestinal tract. Most importantly, we found that patients with antacid treatment had lower uptake both in serum and in tumour-tissue. Thus it seems that antacids hamper the drug absorption. It has recently been shown that absorption and plasma concentration also are largely influenced by food constitutents, and especially milk containing products (Gunnarsson *et al.*, 1990). Thus, it is of clinical importance to consider the use of concomitant drugs and intake of milk-containing food.

The immunohistochemical staining for EMBP, in the present study, showed a high occurrence of EMBP in all astrocytoma patients as shown in an earlier study (von Schoultz *et al.*, 1991). EMBP has been shown to have high affinity binding properties to both EaM and EoM (Forsgren *et al.*, 1979*a,b*) and the anti-tumouros effect of EMP has been suggested to be dependent on the presence of this protein (Björk *et al.*, 1985; Flüchter *et al.*, 1989). Thus, EMBP may play a crucial role in enhancing the cytotoxic action by causing accumulation of especially EaM. The relatively low concentrations of EaM/EoM in the cystic fluid of the tumours compared to the solid tissue component may further indicate the importance of EMBP in the retention of the active metabolites of EMP. On the other hand, in one patient with metastasis from a thyroid carcinoma the staining for EMBP was weak but high levels of EaM and EoM were detected in tumour tissue. This may reflect a high EMBP binding affinity in all tumours for EaM and EoM. However, the exact nature and role of EMBP in brain tumour tissue is so far unknown. The calf brain is shown to have high concentrations of the so called microtubule-associated protein (MAP-2) (Vallee *et al.*, 1982; Caceres *et al.*, 1984). This protein is known to have binding affinity for estramustine and its binding has been suggested to inhibit brain microtubule assembly *in vitro* (Fridén *et al.*, 1987; Stearns & Tew, 1988). Thus, it could be a possibility that EMBP and MAP-2 have common antigenic properties which actually could mean that the EMBP immunohistochemically detected could in fact be MAP-2. Future studies will evaluate this.

In conclusion, the present study demonstrates a high uptake and retention of the metabolites of orally administered EMP in human brain tumour tissue with a high concentration ratio (tumour *vs* serum) especially for the metabolite EaM. Hence, it seems that the blood-brain barrier did not hamper the passage of EMP and/or its metabolites. The presence of EMBP in brain tumour tissue was demonstrated and its importance in retaining the metabolites of EMP was further postulated. These observations may have direct clinical implications, and certainly justify a clinical evaluation of EMP in the treatment of patients with malignant brain tumours.

This study was supported by grants from the Swedish Society Against Cancer (RMC), the Lions Foundation, Jubileumskliniken, Umeå, Sweden, and the Swedish Society for Medical Research. The skilful technical assistance of Ulrika Larsson, Helena Tano, and Arne Norling is acknowledged.

### References

ANDERSSON, S.-B., GUNNARSSON, P.O., NILSSON, T. & PLYMFORS-CHELL, G. (1981). Metabolism of estramustine phosphate in patients with prostatic carcinoma. *Eur. J. Drug. Metab. Pharmacokinet.*, **6**, 149–154.

ANDERSSON, S.-B., LUNDGREN, R. & SVENSSON, L. (1982). Gas chromatographic determination of four metabolites of estramustine phosphate in plasma. *Acta Pharmacol. Swe.*, **19**, 1–10.

BERGH, J., BJÖRK, P., WESTLIN, J.E., BRODIN, O. & NILSSON, S. (1988). Expression of estramustine-binding protein (EMBP) in human lung cancer cell lines. *Cancer Res.*, **48**, 4615–4619.

BJÖRK, P., FRITJOFSSON, Å. HARTLEY-ASP, B. (1985). Uptake and binding of estramustine and estromustine, metabolites of estramustine phosphate (Estracyt), in the human prostate, and new aspects on the cytotoxic activity of estramustine phosphate in vitro. In *Experimentelle Urologie*, Harzmann, R. (ed) pp. 341–353. Springer Verlag: Berlin-Heidelberg.

CACERES, A., BINDER, L.I., PAYNE, M.R., BENDER, P., REBHUN, L. & STEWARD, O. (1984). Differential subcellular localization of tubulin and the microtubule-associated protein MAP-2 in brain tissue as revealed by immunocytochemistry with monoclonal hybridoma antibodies. *J. Neurosci.*, **4**, 394–410.

FLÜCHTER, S.H., NELDE, H.J., BJÖRK, P., MÜNTZING, J. & BICHLER, K.-H. (1989). Effects of treatment on the expression of estramustine-binding protein (EMBP) in prostatic cancer patients: an immunohistochemical study. *The Prostate*, **14**, 27–43.

FORSGREN, B., BJÖRK, P., CARLSTRÖM, K., GUSTAFSSON, J.-Å., POUSETTE, Å. & HÖGBERG, B. (1979a). Purification and distribution of a major protein in rat prostrate that binds to estramustine, a nitrogen mustard derivate of estradiol-17$\beta$. *Proc. Nat. Acad. Sci.*, **76**, 3149–3153.

FORSGREN, B., GUSTAFSSON, J.-Å., POUSETTE, Å. & HÖGBERG, B. (1979b). Binding characteristics of a major protein in rat prostate cytosol that interacts with estramustine, a nitrogen mustard derivate of 17$\beta$-estradiol. *Cancer Res.*, **39**, 5155–5164.

FRIDÉN, B., WALLIN, M., DEINUM, J., PRASADE, V. & LUDUENA, R. (1987). Effect of estramustine-phosphate on the assembly of trypsin-treated microtubules and microtubules reconstituted from purified tubulin with either tau, MAP-2 or the tubulin fragment of MAP-2. *Arch. Biochem. Biophys.*, **257**, 123–130.

GUNNARSSON, P.O., ANDERSSON, S.-B., JOHANSSON, S.-Å., NILSSON, T. & PLYM-FORSHELL, G. (1984). Pharmacokinetics of estramustine phosphate in prostatic cancer patients. *Eur. J. Clin. Pharmacol.*, **26**, 113–119.

GUNNARSSON, P.O., DAVIDSSON, T., ANDERSSON, S.-B., BACKMAN, C. & JOHANSSON, S.-Å. (1990). Impairment of estramustine phosphate absorption by concurrent milk and food intake. *Eur. J. Clin. Pharma-col.*, **38**, 189–193.

HARTLEY-ASP, B. (1984). Estramustine-induced mitotic arrest in two human prostatic carcinoma cell lines DU 145 and PC-3. *The Prostate*, **5**, 93–100.

VON HOLST, H., KNOCHENHAUER, E., BLOMGREN, H., COLLINS, V.P., EHN, L., LINDQUIST, M., NORÉN, G. & PETERSON, C. (1990). Uptake of adriamycin in tumour and surrounding brain tissue in patients with malignant gliomas. *Acta Neurochir. (Wien)*, **104**, 13–16.

HSU, S.M., RAINE, L. & FANGER, H. (1981). Use of Avidin-Biotin-Peroxidase complex (ABC) in immunoperoxidase tech-niques: A comparison between ABC and unlabeled antibody (PAP) procedures. *J. Histochem, Cytochem.*, **29**, 577–580.

KRUSE, E. & HARTLEY-ASP, B. (1988*a*). Uptake and metabolism of estramustine in the Dunning R3327H tumour. *In Vivo*, **2**, 371–376.

KRUSE, E., JOHANSSON, S.-Å., HARTLEY-ASP, B. & GUNNARSSON, P.O. (1988*b*). Distribution and metabolism of estramustine in HeLa cells and the human prostatic tumour cell line 1013L. *Biochem. Pharmacol.*, **37**, 3161–3167.

NORLÉN, B.J., ANDERSSON, S.-B., BJÖRK, P., GUNNARSSON, P.O. & FRITJOFSSON, Å. (1988). Uptake of estramustine phosphate (Estracyt) metabolites in prostatic cancer. *J. Urology*, **140**, 1058–1062.

VON SCHOULTZ, E., LUNDBLAD, D., BERGH, J., GRANKVIST, K. & HENRIKSSON, R. (1988). Estramustine binding protein and antiproliferative effect of estramustine in human glioma cell lines. *Br. J. Cancer*, **58**, 326–329.

VON SCHOULTZ, E., GUNNARSSON, P.O. & HENRIKSSON, R. (1989). Uptake, metabolism and antiproliferative effect of estramustine phosphate in human glioma cell lines. *Anticancer Res.*, **9**, 1713–1716.

VON SCHOULTZ, E., LUNDGREN, E. & HENRIKSSON, R. (1990). Effects of estramustine and its constituents on human malignant glioma cells. *Anticancer Res.*, **10**, 693–696.

VON SCHOULTZ, E., BERGENHEIM, T., GRANKVIST, K.P. & HENRIKSSON, R. (1991). Estramustine binding protein in human brain tumour tissue. *J. Neurosurg.*, **74**, 962–964.

STEARNS, M. & TEW, K.D. (1988). Estramustine binds MAP-2 to inhibit microtubule assembly *in vitro*. *Cell Sci.*, **89**, 331–342.

STERNBERGER, L.A. (1979). The unlabelled antibody peroxidase-antiperoxidase (PAP) method. In *Immunochemistry*, 2nd edition, p 104, John Wiley & Sons: New York.

VALLEE, R.B. (1982). A taxol-dependent procedure for the isolation of microtubules and microtubule-associated proteins. *J. Cell Biol.*, **92**, 435–442.

WHITTLE, I.R., MACPHERSON, J.S., MILLER, J.D. & SMYTH, J.F. (1990). The disposition of TCNU (tauromustine) in human malignant glioma: pharmacokinetic studies and clinical implications. *J. Neurosurg.*, **72**, 721–725.